# Oxygen vacancy clusters essential for the catalytic activity of CeO_2_ nanocubes for o-xylene oxidation

**DOI:** 10.1038/s41598-017-13178-6

**Published:** 2017-10-09

**Authors:** Lian Wang, Yunbo Yu, Hong He, Yan Zhang, Xiubo Qin, Baoyi Wang

**Affiliations:** 1 State key Joint Laboratory of Environmental Simulation and Pollution Control, Research Center for Eco-Environmental Sciences, Chinese Academy of Sciences, Beijing, 100085 China; 2 Center for Excellence in Regional Atmospheric Environment, Institute of Urban Environment, Chinese Academy of Sciences, Xiamen, 361021 China; 3 University of Chinese Academy of Sciences, Beijing, 100049 China; 4 Key Laboratory of Nuclear Analysis Techniques, Institute of High Energy Physics, Chinese Academy of Sciences, Beijing, 100049 China

## Abstract

Catalytic oxidation of o-xylene was investigated on CeO_2_ nanocubes calcined at 350, 450, 550, and 650 °C, among which the samples calcined at 550 °C exhibited the highest activity and long durability. Positron annihilation spectroscopy measurements revealed that the size and distribution of oxygen vacancies for CeO_2_ nanocubes could be tuned by carefully controlling the calcination temperature. An excellent linear correlation between a factor related to size and density of oxygen vacancy clusters and reaction rate of o-xylene oxidation was revealed on ceria nanocubes. This means that oxygen vacancy clusters with suitable size and distribution are responsible for catalytic reaction via simultaneous adsorption and activation of oxygen and o-xylene. Electron spin resonance spectra revealed that over the CeO_2_ cubes, water vapor significantly promoted the formation of ∙OH radicals with a sharp decrease in the signals relating to oxygen vacancies, accelerating the transformation of o-xylene to the intermediate benzoate species, resulting in an enhancement of catalytic activity. Water thus serves as a “smart” molecule; its introduction into the feed mixture further confirmed the key role of oxygen vacancies in the catalytic performance of CeO_2_ nanocubes. A possible mechanism of oxygen vacancy formation during the calcination process was also proposed.

## Introduction

Volatile organic compounds (VOCs), emitted from human activities such as industrial processes and vehicle exhausts, are recognized as hazardous air pollutants due to their toxicity and role as secondary air pollution precursors^1–3^. Typically, BTX (benzene, toluene, xylene) have received a great deal of attention because of their potential risks to human health^3,4^. It is therefore very important to develop efficient techniques for BTX abatement from polluted air. To this aim, catalytic oxidation of BTX by noble metal and metal oxide catalysts has been widely studied^4–6^. Often, noble metal catalysts are more active than other metal oxide catalysts, while the latter are usually low-cost. Thus, many efforts have been devoted to developing non-previous-metal catalysts with excellent activity.

For a metal oxide catalyst, the intrinsic properties of metal cations and oxygen anions are just the two sides of the same coin^7,8^. With this in mind, there are two strategies or opinions on the design of metal oxides with high catalytic performance. Firstly, increasing the richness of active surface cationic sites on metal oxide has been successfully realized through morphology-controlled synthesis, thus creating nanocatalysts with excellent activity^9^. On the other hand, surface oxygen vacancies are regarded as the most reactive sites in catalysis^10,11^, and thus extraction of oxygen anions from metal oxides to produce oxygen vacancies can also significantly boost the chemical reactions occurring on the metal oxide catalysts^12^.

It is well known that oxygen vacancies dominate the electronic and redox properties of ceria (CeO_2_), leading to its an important role in catalysis such as in vehicle exhaust after-treatment, water-gas shift reactions, fuel cells, CO oxidation, and production and purification of hydrogen^13–16^. Over CeO_2_, meanwhile, oxygen vacancies were found to promote the decomposition of water molecules into active ∙OH radicals, providing the opportunity to develop an active catalyst in the presence of water vapor^11^. CeO_2_-based catalysts have also been employed for BTX removal, the design of which has mostly focused on the relationship between their reducibility and catalytic activity^17–22^. More recently, our study showed that nanosized CeO_2_ particles, cubes, and rods exhibit high activity for o-xylene oxidation which is similar to that of noble metal catalysts, while being much higher than that of noble-metal-free catalysts reported elsewhere^23^. Over these nanoceria materials with well-defined facets, it is interesting that oxygen vacancy clusters (VCs) play a key role in o-xylene oxidation. It was also found that the activity of CeO_2_ cubes was enhanced by the presence of water vapor. The apparent water-tolerance of these nanocubes is highly desirable for their application, since water vapor is inevitable in various exhausts.

To further reveal the intrinsic properties of nanoceria for o-xylene oxidation, herein, cubic CeO_2_ was prepared by a hydrothermal method, while the nature of oxygen vacancies was tuned by carefully controlling the calcination temperature. It was found that oxygen vacancy clusters with suitable size and/or structure are essential for catalytic reaction via simultaneous adsorption and activation of oxygen and o-xylene molecules. Also, oxygen vacancies play a crucial role in the activation and decomposition of water molecules to produce ∙OH radicals, thus relating to the enhancement of o-xylene oxidation over CeO_2_ cubes by water vapor.

## Results and Discussion

### Structural features of CeO_2_ nanocubes

**Figure 1:**
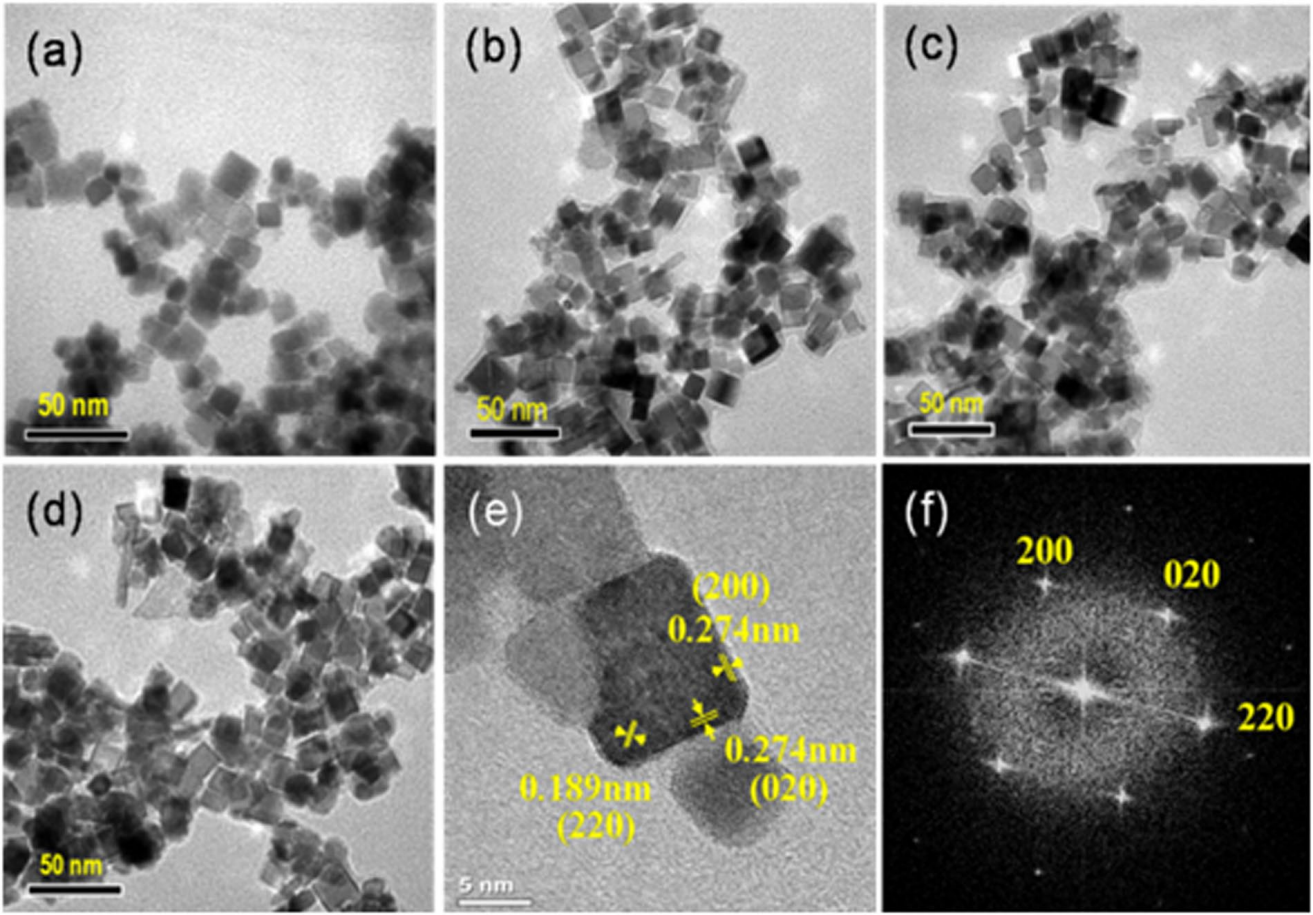
TEM images of CeO_2_ nanocubes calcined at 350 °C (**a**), 450 °C (**b**), 550 °C (**c**), 650 °C (**d**); HRTEM image of CeO_2_ nanocubes calcined at 550 °C (**e**); fast Fourier transform (FFT) analysis of CeO_2_ nanocubes calcined at 550 °C (**f**). Scale bar: 50 nm for a–d, 5 nm for e.

As shown by XRD measurements (Figure S1), the typical diffraction peaks of the pure fluorite cubic structure were observed for all CeO_2_ nanocubes calcined at different temperatures (JCPDS 34–0394). The fluorite phase of CeO_2_ was also observed by Raman spectroscopy (Supporting Information, Figure S2), exhibiting a strong band at 460–463 cm^−1^ ^24,25^. The sample calcined at 350 °C exhibited a surface area of 78 m^2^·g^−1^ (Table 1). Calcination of the sample at 450 °C resulted in a slight increase in the surface area if compared with that calcined at 350 °C. As listed in Table 1, however, further increasing the calcination temperature lowered the surface area. TEM images (Fig. 1a–d) revealed that the calcined samples exhibit cubic morphology with a uniform size. The HRTEM images in Fig. 1e (and Figure S3) combined with FTT analysis (Fig. 1f) display the clear (200) and (220) lattice fringes with the interplanar spacings of 0.274 and 0.189 nm, respectively, implying that the CeO_2_ nanocubes are enclosed by {100} planes^26^.

**Table 1:** Physicochemical properties and o-xylene oxidation rates and orders over CeO_2_ nanocubes calcined at different temperatures.

Sample (°C)^a^	BET area (m^2^·g^−1^)	Reaction rate (10^−3^ μmol·s^−1^m^−2^)^b^	Reaction order of O_2_^b^	Reaction order of o-xylene^b^
350	78	1.5	0.33	0.94
450	83	1.8	0.29	0.36
550	59	4.0	0.23	0.33
650	43	2.9	0.19	0.53

^a^Calcination temperature;

^b^o-xylene conversion kept below 15% at 230 °C by varying the space velocity to realize the differential-reactor assumption.

### Catalytic activity of CeO_2_ nanocubes for o-xylene oxidation

**Figure 2:**
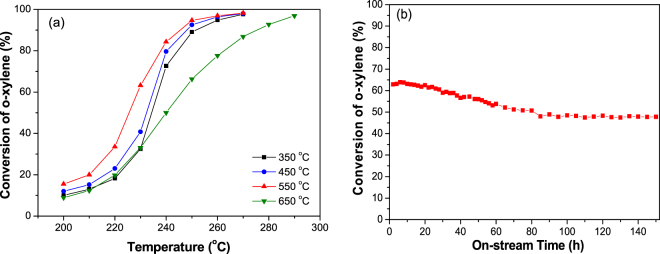
(**a**) The catalytic activity of CeO_2_ nanocubes calcined at different temperatures for o-xylene oxidation; (**b**) durability of CeO_2_ calcined at 550 °C for o-xylene oxidation at 230 °C. Reaction conditions: catalyst weight 100 mg, o-xylene 500 ppm, 20 vol% O_2_, N_2_ balance, total flow rate = 100 mL/min, WHSV = 60,000 mL·h^−1^·g^−1^.

Figure 2 shows the catalytic activities of CeO_2_ calcined at different temperatures for o-xylene oxidation. For the sample calcined at 350 °C, 10% conversion of o-xylene was obtained at 200 °C (Fig. 2a). With increasing reaction temperature, the catalytic oxidation of o-xylene was promoted sharply, giving 50% conversion at 234 °C. After this, the o-xylene conversion increased gradually as temperature increased, showing 90% conversion and complete oxidation at 250 and 270 °C, respectively. Compared with the sample calcined at 350 °C, the CeO_2_ calcined at 450 °C always showed higher activity for o-xylene oxidation at a given reaction temperature. The sample calcined at 550 °C exhibited much higher activity for o-xylene oxidation than those calcined at other temperatures (Fig. 2a and Table 1), for which the specific rate at 230 °C was 0.004 μmol∙s^−1^ m^−2^, 1.7 times higher than that calcined at 350 °C. Catalytic oxidation of o-xylene was also carried out continuously at 230 °C over the CeO_2_ nanocubes calcined at 550 °C, during which ca 50% conversion was maintained over the whole time range of 150 h, demonstrating a high level of durability (Fig. 2b).

**Figure 3:**
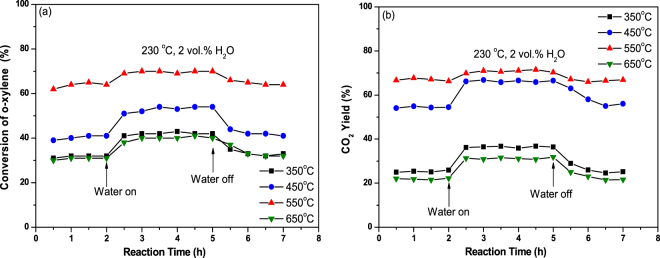
Influence of water vapor on the activity of CeO_2_ calcined at different temperatures (**a**) for o-xylene conversion and (**b**) CO_2_ yield at 230 °C. Reaction conditions: catalyst weight 100 mg, o-xylene 500 ppm, 20 vol% O_2_, N_2_ balance, total flow rate = 100 mL/min, WHSV = 60,000 mL·h^−1^·g^−1^.

The change in catalytic activity of CeO_2_ nanocubes induced by water vapor addition was evaluated at the reaction temperature of 230 °C. In the absence of water vapor, o-xylene conversion remained at 32% over the CeO_2_ calcined at 350 °C (Fig. 3a). When 2 vol% water vapor was introduced into the feed gas, an increase in the o-xylene conversion of 10% was observed. As shown in Fig. 3b, simultaneously, the CO_2_ yield showed almost the same tendency for increase (growth of 11%), indicating that water vapor enhances the complete oxidation of o-xylene. When water vapor was removed from the gas mixture, the o-xylene conversion recovered to its initial level as that without water vapor. Such reversible effects of water vapor on o-xylene conversion and CO_2_ yield were also clearly observed over the other samples.

### Oxygen species and vacancies on the surface of CeO_2_ nanocubes

XPS results demonstrated that there was Ce^3+^ existed in all the CeO_2_ nanocubes calcined at different temperatures (Supporting Information, Figure S4a,b and corresponding description), indicating the generation of oxygen vacancies^27^. Using XPS measurements performed under ultra-high vacuum (UHV), Xu and co-workers found that the Ce^3+^ concentration was almost the same for CeO_2_ samples with particle sizes ranging from 4.4–9.9 nm, which was not in agreement with the amount of surface oxygen species determined by a TPR experiment^28^. Ambient-pressure XPS measurements, however, have shown that the fraction of Ce^3+^ in ceria-based samples is often related to the density of oxygen vacancies^29–31^. In our case, the XPS experiment was performed under UHV, thus the Ce 3d XPS results were only used to confirm the presence of Ce^3+^ and oxygen vacancies, not to quantitatively analyze their concentrations in CeO_2_ samples calcined at different temperatures. The existence of oxygen vacancies was also confirmed by the appearance of defect-induced mode bands in the Raman spectra at around 602 cm^−1^ ^25^, which means that active sites are available for oxygen adsorption and activation in the process of catalytic oxidation. Pfau and Schierbaum concluded that the formation of Ce^3+^-related surface defects leads to an O1s core level peak with a shift of 2.4 eV to higher binding energies as compared with the low energy O1s core level^32^. Wang *et al*. proposed that the peak at binding energy 529.01 eV can be attributed to lattice oxygen ions in CeO_2_, the peak at 530.13 eV to absorbed oxygen, and the peak at 531.33 eV to lattice oxygen ions in Ce_2_O_3_^33^. Thus, the peak in the O1s spectra at 531.5 eV, which was attributed to surface oxygen, should include a certain content of Ce^3+^-related surface defects, i.e. oxygen vacancies. The higher ratio of surface oxygen, the higher oxygen vacancy level is. Therefore, the CeO_2_ samples calcined at 450 and 550 °C possess larger amounts of oxygen vacancies (Figure S4c and Table 2).

**Table 2:** XPS results for CeO_2_ nanocubes calcined at different temperatures.

Sample (°C)^a^	Concentration of Ce^3+^ (%)	BE (eV)		O_sur_/(O_latt_ + O_sur_) (%)
		O_latt_	O_sur_	
350	21.1	529.1	531.7	15.4
450	22.3	529.1	531.4	19.2
550	21.2	529.0	531.5	16.1
650	20.5	528.9	531.6	11.5

^a^Calcination temperature.

Positron annihilation spectroscopy (PAS) is a well-established technique to detect atomic defects in solid materials. In this case, the lifetime of positrons depends on the electron density at the annihilation site: the lower the electron density, the longer the lifetime of positrons is. As a result, the size and intensity of defects in a solid can be revealed^34^. Three lifetime values (τ_1_, τ_2_, and τ_3_) were clearly observed for all the CeO_2_ nanocubes calcined at different temperatures, the relative intensities of which were denoted as I_1_, I_2_, and I_3_, respectively (Table 3 and Figure S5). The longest value, τ_3_, the so-called bulk lifetime, was assigned to the annihilation of ortho-positronium atoms in the large voids. For all the samples, the intensity of the bulk lifetime peak (I_3_) was the lowest and hardly changed with calcination temperature, suggesting that few large voids were present in any of the samples. Based on the previous studies^35–38^, the shortest one (τ_1_) was due to free annihilation of positrons (denoted as FA hereafter) or positrons trapped by single oxygen vacancies (denoted as SV hereafter). The middle one (τ_2_) could be assigned to positrons captured by surface oxygen vacancy clusters (denoted as VCs hereafter, i.e. dimmers, trimers, or larger clusters)^35–38^. It is interesting to note that increasing the calcination temperature from 350 to 450 °C significantly increased the value of τ_2_, indicating the formation of larger size VCs. Further increase in calcination temperature to 550 °C hardly changed the size of VCs while increasing their concentration. The sample calcined at 650 °C exhibited the largest VCs at the expense of lowering their concentration. To further describe the feature of the overall defects status, the average lifetime (τ_av_) was calculated as follows^23^:

1 $${{\rm{\tau }}}_{{\rm{av}}}={{\rm{\tau }}}_{1}\cdot {{\rm{I}}}_{1}+{{\rm{\tau }}}_{2}\cdot {{\rm{I}}}_{2}+{{\rm{\tau }}}_{3}\cdot {{\rm{I}}}_{3}$$

**Table 3:** Position lifetime parameters of CeO_2_ nanocubes calcined at different temperatures.

Sample (°C)^a^	τ_1_ (ps)	τ_2_ (ps)	τ_3_ (ns)	I_1_ (%)	I_2_ (%)	I_3_ (%)	τ_av_ (ps)^b^
350	181 ± 3.2	340 ± 3.1	2.79 ± 0.10	41.51 ± 0.81	57.09 ± 0.80	1.39 ± 0.04	308 ± 8.6
450	190 ± 1.8	420 ± 0.7	3.00 ± 0.06	43.84 ± 0.28	54.91 ± 0.28	1.25 ± 0.02	351 ± 4.2
550	183 ± 4.2	414 ± 2.9	2.53 ± 0.05	36.72 ± 0.88	61.82 ± 0.87	1.46 ± 0.04	360 ± 10.4
650	189 ± 3.1	434 ± 3.9	2.50 ± 0.07	43.71 ± 0.84	54.76 ± 0.83	1.53 ± 0.05	359 ± 11.1

^a^Calcination temperature;

^b^τ_av_ = τ_1_ I_1_ + τ_2_ I_2_ + τ_3_ I_3_.

As shown in Table 3, the value of τ_av_ increases with increasing calcination temperature, also indicating that calcinations of samples at high temperature enhances the formation of defects in nanoceria.

### Relationship between structure of CeO_2_ nanocubes and activity for o-xylene oxidation

**Figure 4:**
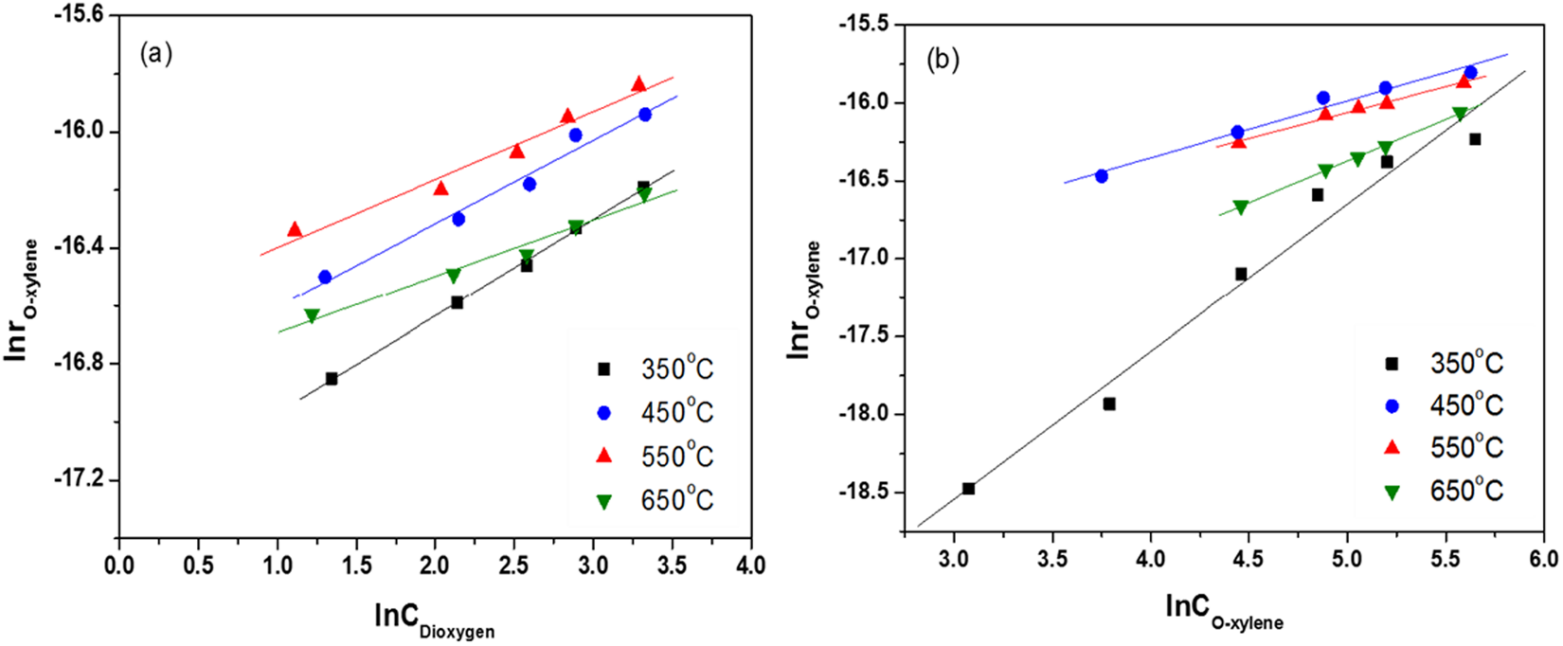
(**a**) Rates of o-xylene oxidation as a function of O_2_ over CeO_2_ nanocubes. The reaction rates were measured at 230 °C. The concentration of O_2_ was varied in the range of 5–30 vol% and the concentration of o-xylene was 200 ppm. The o-xylene conversion was adjusted to below 15% by varying the space velocity. (**b**) Rates of o-xylene oxidation as a function of o-xylene concentration over CeO_2_ nanocubes. The reaction rates were measured at 230 °C. The concentration of o-xylene was varied in the range of 25–300 ppm and the concentration of O_2_ was 20 vol%. The o-xylene conversion was adjusted to below 15% by varying the space velocity.

Figure 4a and Table 1 show the dependence of o-xylene oxidation rate on the concentration of O_2_ over CeO_2_ calcined at different temperatures. Over the sample calcined at 350 °C, the reaction order of O_2_ was 0.33, suggesting that large amounts of O_2_ were adsorbed on the surfaces of CeO_2_, resulting in a weak dependency of the reaction rate on O_2_ concentration. Increasing the calcination temperature gradually decreased the reaction order for O_2_, giving the values of 0.29, 0.23, 0.19 for the samples calcined at 450, 550, 650 °C, respectively. As shown in Table 1 and Fig. 4b, the lowest reaction order of o-xylene (0.33) was achieved for CeO_2_ calcined at 550 °C, which also indicates that the largest amount of o-xylene was adsorbed on the surfaces of this sample. On the CeO_2_ calcined at 350 °C, however, the order of o-xylene was rose to 0.94, indicative of a weak interaction.

Generally, the steric effect is a crucial factor governing the kinetics of chemical reactions. For a given molecule, its cross-sectional area or volume can be used as a metric for the steric effect. Using a space-filling model (CPK model)^39,40^, the cross-sectional areas of O_2_ and o-xylene were calculated to be 0.167 and 0.626 nm^2^, respectively. As a result, it is reasonable that, on a given sample, the reaction order of o-xylene is always higher than that of O_2_. In other words, the adsorption and activation of o-xylene on the surface was more difficulty than that of O_2_. On the sample calcined at 350 °C, the VCs exhibit the smallest size. This possibly indicates that the active sites are not easily accessible for o-xylene adsorption, then relating to a high reaction order for o-xylene (0.94). The CeO_2_ calcined at 650 °C possessed VCs with the biggest size, resulting in the strongest ability for O_2_ adsorption (giving the lowest O_2_ order of 0.19), thus suppressing the adsorption of o-xylene (giving the o-xylene order of 0.53). Such adsorption behavior of reactants may lower the catalytic performance of samples calcined at 650 °C since highly catalytic activity requires proper adsorption ability for both o-xylene and O_2_ on the catalyst surface according to the Sabatier principle^41^. As for the sample calcined at 550 °C, it should be noted that, the reaction order of o-xylene was lower than for those calcined at other temperatures, indicating an advantage in adsorption and activation of o-xylene.

As mentioned above, oxygen vacancies dominate the electronic and chemical properties of ceria, playing an important role in catalysis. Recently, it has been reported that CeO_2_ nanorods with predominantly exposed (111) and (100) planes exhibited higher activity for CO oxidation than those enclosed by (110) and (100) planes, originating from a larger amounts of oxygen vacancy clusters in the former^35^. Recently, Lawrence *et al*. found that nanosized CeO_2_ rods and particles subjected to a low-pressure thermal pretreatment possessed a high density of oxygen vacancy defects, promoting CO oxidation at low temperatures^31^. If oxygen vacancies actually play a role in the catalytic oxidation of o-xylene, a relationship between the vacancies and the reaction rate of o-xylene should exist. To further identify which kinds of oxygen vacancies (single oxygen vacancy and aggregates) are essential for the catalytic oxidation of o-xylene, a variable (hereafter denoted as the Vac factor, in which the size and relative density of oxygen vacancies both contribute to the catalytic reaction) was defined as follows:

2 $${\rm{Vac\; factor}}={{\rm{\tau }}}_{{\rm{i}}}\cdot {{\rm{I}}}_{{\rm{i}}}/{{\rm{\tau }}}_{{\rm{av}}}(i=1,2)$$

where, τ_i_ and I_i_ are lifetimes of positrons in SV or FA (or VCs) and the corresponding intensity, respectively; τ_av_ is the average lifetime of the positron.

**Figure 5:**
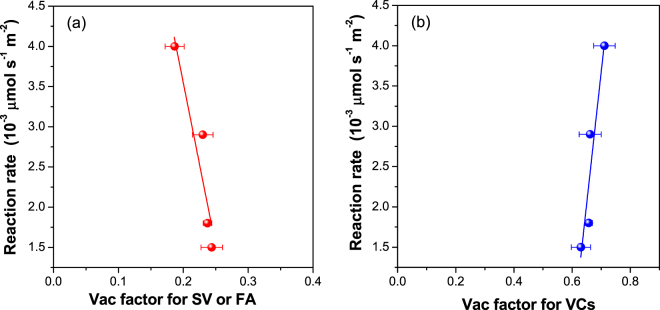
(**a**) The relationship between the Vac factor for single oxygen vacancies (SV) or free annihilation of positrons (FA) and reaction rate of o-xylene, and (**b**) the relationship between the Vac factor for oxygen vacancy clusters (VCs) and reaction rate of o-xylene over ceria nanocubes calcined at different temperatures.

Surprisingly, there is a negative linear correlation between the Vac factor of SV or FA and reaction rate of o-xylene (Fig. 5a), while a positive linear correlation between the Vac factor of VCs and reaction rate can be drawn for ceria calcined at different temperatures (Fig. 5b). Qualitatively, this result identifies that it is the VCs that determine the activity of ceria for the catalytic oxidation of o-xylene, during which the size and relative density of VCs both contribute to the catalytic reaction.

Driven by a gain in energy during agglomeration, oxygen vacancies on the ceria surface are prone form clusters^42–44^. As a result, VCs exhibit higher relative intensity (Table 3). The DFT+U calculation performed by Jiang and Dai indicated that two neighboring single oxygen vacancies working together would be more efficient for O_2_ activation than those working alone on the Co_3_O_4_(110) surface^7^. Such a theoretical result possibly predicts that the oxygen vacancy clusters in metal oxides possess higher catalytic activity than single oxygen vacancies. By using quantitative temporal analysis of products (TAP) techniques, it was found that the activity of Au/CeO_2_ for CO oxidation was significantly enhanced by the removal of about 7% of the surface oxygen, while over-reduction led to lower activity^45^. By using a pulse CO analysis, a volcano-like relationship between the amount of oxygen vacancies and the activity of Ir-in-CeO_2_ for CO oxidation was established^46^. In that case, the concentration of oxygen vacancies was calculated based on the released amount of CO_2_ and correspondingly the accumulation of carbonates with pretreatment by pulses of CO, thus it was difficult to reveal the structural features of oxygen vacancies such as the size and distribution. Our results confirm that oxygen vacancy clusters were predominant for the CeO_2_ cubes. A linear relationship between the value of τ_2_∙I_2_/τ_av_ and the reaction rate of o-xylene indicates that both the size and relative density of VCs contributed to the activity of the CeO_2_ cubes. The sample calcined at the temperature of 550 °C exhibited the highest activity, indicating that oxygen vacancy clusters with suitable size and distribution are essential for catalytic reaction via simultaneous adsorption and activation of oxygen and o-xylene molecules.

### Water vapor effect on the pathway of o-xylene oxidation over CeO_2_ nanocubes

**Figure 6:**
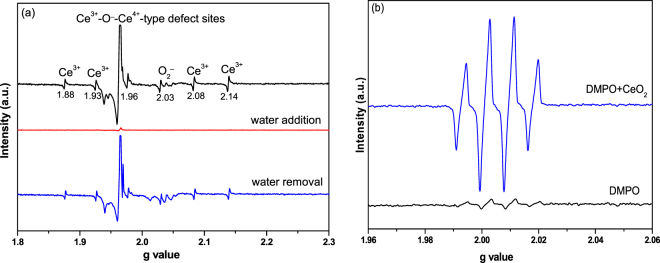
(**a**) EPR profiles measured at room temperature for CeO_2_ nanocubes calcined at 550 °C with (100 mg CeO_2_ + 0.05 mL deionized H_2_O) or without water; (**b**) DMPO spin-trapping ESR profiles recorded at ambient temperature in an aqueous dispersion of CeO_2_ nanocubes (10 g/L) calcined at 550 °C (for DMPO-OH^•^ adduct).

As o-xylene oxidation occurred on the surface of CeO_2_ cubes, another interesting finding is that the presence of water vapor promoted the complete conversion of o-xylene to CO_2_. To reveal how such enhancement is triggered by water vapor, ESR analysis was performed without or with water vapor (Fig. 6a). In the absence of water vapor, Ce^3+^, O_2_^−^ species, and Ce^3+^-O-^−^Ce^4+^-type defect sites were distinctly observed on the sample calcined at 550 °C^47–49^, further confirming the results of XPS and PAS. Introduction of water vapor resulted in the disappearance of the characteristic signals due to Ce^3+^, oxygen vacancies, and active oxygen species, suggesting that some reactions occurred among these species/sites and water molecules. Based on previous studies^50,51^, one possibility that could be speculated is the formation of ∙OH radicals. To test this hypothesis, ESR spectra of the DMPO-OH^•^ spin adduct were measured after mixing the CeO_2_ powders with DMPO solution at room temperature since ∙OH radicals are very unstable in water (Fig. 6b). The four characteristic peaks of the DMPO-OH^•^ species, a 1:2:2:1 quartet pattern, were clearly observed, confirming the production of ∙OH radicals after adding water to the surface of CeO_2_^50,51^. Numerous studies have been performed on the process of water adsorption on ceria, and it is well accepted that H_2_O molecules strongly and dissociatively bind on oxygen vacancy sites^11^. Thus, it is possible that the formation of ∙OH radicals is dependent on the activation of H_2_O molecules on the oxygen vacancy sites through Ce^3+^/Ce^4+^ redox cycle^11,50,51^. Generally, ∙OH radicals possess high oxidation ability, and thus relating to the promoting effect of water vapor on complete oxidation of o-xylene to CO_2_. After removal of water vapor from the sample by heating the sample at 150 °C (Fig. 6a), it should be noted that the signals assignable to Ce^3+^, O_2_^−^, and Ce^3+^-O-^−^Ce^4+^-type defect sites appeared again and the sample recovered its initial reactivity level in the absence of water vapor. Such a reversible effect is in good agreement with the results of activity measurement presented in Fig. 3.

**Figure 7:**
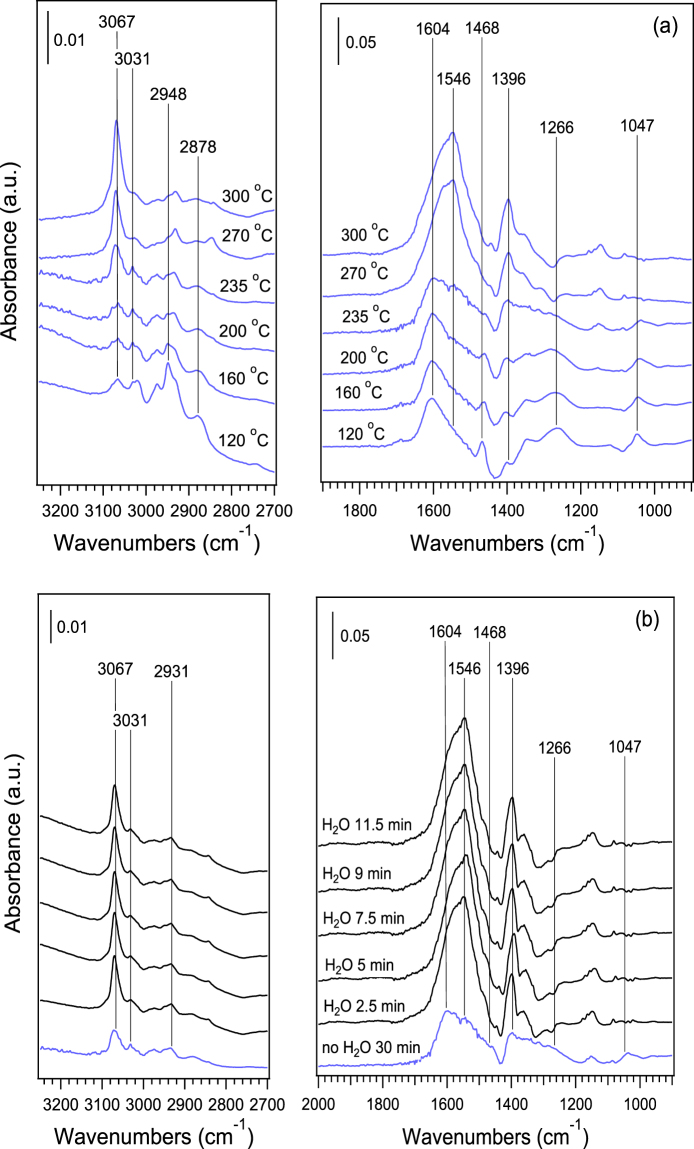
*In situ* DRIFTS spectra of CeO_2_ nanocubes calcined at 550 °C during exposure to a flow of 500 ppm o-xylene + 80 mL/min N_2_ + 20 mL/min O_2_ at different temperatures without water vapor (**a**), and with water vapor addition at 235 °C (**b**).

To investigate the pathway of o-xylene oxidation over the CeO_2_ nanocubes, *in situ* DRIFTS experiments were performed. Figure 7a shows the DRIFT spectra of CeO_2_ calcined at 550 °C during exposure to a flow of 500 ppm o-xylene in 20 vol% O_2_. At temperatures below 235 °C, four peaks attributed to the stretching vibrations of C-H bonds were observed. Among them, the bands at 3067 and 3031 cm^−1^ were assigned to the phenylic C-H stretching vibration (Table S2)^52,53^. The bands at 2948 and 2878 cm^−1^ are typically due to the symmetric and antisymmetric stretching vibrations of C-H bonds of benzyl species. The intensity of bands at 3031, 2948, and 2878 cm^−1^ decreased with increasing reaction temperature, and almost disappeared at 300 °C. Simultaneously, however, the intensity of the high-frequency band at 3067 cm^−1^ increased, indicating the transformation of the methyl group of the o-xylene molecule^52,53^. Within the range of 1800–1000 cm^−1^, the bands related to ring vibrations of aromatic systems were clearly observed at 1604 and 1468 cm^−1^, the intensity of which exhibited a gradual decrease as temperature increased from 120 °C to 235 °C, indicating a change in the electronic environment of the aromatic ring. Within this temperature range, meanwhile, an increased intensity of the characteristic bands attributed to the carboxylate group appeared at 1546 and 1396 cm^−1^, suggesting the formation of benzoate species^52,53^. Further heating of the sample to 300 °C resulted in a disappearance of the two bands due to aromatic rings, the occurrence of which was companied by a sharply increased intensity of peaks for benzoate species. In addition, peaks at 1266 and 1047 cm^−1^ could be assigned to o-xylene, the intensity of which decreased with increasing temperature, and disappeared above 235 °C^54^. Obviously (Fig. 7b), the introduction of water vapor distinctly decreased the intensity of bands at 1604 and 1468 cm^−1^ while significantly increasing the intensity of bands at 1546 and 1396 cm^−1^. These results strongly suggested that the presence of water vapor promoted the formation of benzoate at the expense of o-xylene.

Previous studies carried out within the temperature range of 450–1000 K showed that the gas-phase reaction of o-xylene with ∙OH radicals exhibited an Ea value of 0.29 kJ/mol^55,56^, which is also indicative of a low reaction energy barrier if this reaction occurs on the surface of catalysts. As indicated by Fig. 6, the introduction of water vapor triggered the formation of ∙OH radicals via dissociation of H_2_O on oxygen vacancy sites of CeO_2_. As a result, it is reasonable that the conversion of o-xylene over CeO_2_ nanocubes was enhanced by water vapor (Figs 3 and 7). Using *in situ* DRIFTS measurement, Zhao *et al*.^53^ revealed that benzoate species are the main intermediates in the catalytic oxidation of toluene, which should be further oxidized by O_2_ to the final products of CO_2_ and H_2_O. Figure 7 shows that adsorbed o-xylene predominated on the surface of CeO_2_ nanocubes at temperatures below 235 °C, while at temperatures above 235 °C, only benzoate was observed during the catalytic oxidation of o-xylene. With this in mind, it is possible that the pathway of o-xylene oxidation over CeO_2_ nanocubes can be regarded as follows:

3 $$o \mbox{-} \mathrm{xylene}+{{\rm{O}}}_{{\rm{2}}}\to {\rm{benzoate}}\to {{\rm{CO}}}_{{\rm{2}}}+{{\rm{H}}}_{{\rm{2}}}{\rm{O}}$$

Introduction of water vapor into the feed of o-xylene + O_2_ promoted the formation of benzoate and its further oxidation towards CO_2_. As a result, increased o-xylene conversion and CO_2_ yield were obtained in the presence of water vapor (Fig. 3).

There have been seminal papers on ceria shapes and ceria oxygen vacancies, particularly on the atomic surface structures of CeO_2_ nanocubes^57–60^. By using HRTEM and three-dimensional electron tomography, Kaneko *et al*.^57^ found that CeO_2_ nanocubes exposed predominantly {200} facets, with truncation of the corners exposing {111} facets. This structural feature of CeO_2_ nanocubes was further revealed by clearly imaging the O atoms^60^. More importantly, it was found that, although {111} corners are present on the CeO_2_ nanocubes, the contribution of these sites to the total adsorption of methoxy species should be negligible. A previous study confirmed that methanol can act as a “smart” molecule for probing the nature of the surface sites of ceria catalysts^59^. It dissociates on the surface to form methoxy species whose structure is dependent on the nature of the surface sites, i.e., the coordination status of surface Ce cations and the presence of oxygen vacancies. As a result, Lin and co-workers concluded that these mixed surface terminations described above should be intrinsic to the pristine {100} surfaces of the nanocubes^60^.

As shown in Fig. 1 and Figure S3, the CeO_2_ nanocubes calcined at low temperatures of 350 and 450 °C were clearly enclosed by {100} facets, while truncated corners appeared on the sample calcined at 550 °C. This means that new facets composed of (111) surfaces were present at the corners of the sample calcined at 550 °C. More recently, our results confirmed that the influence of water vapor on the activity of nanosized CeO_2_ calcined at the same temperature of 450 °C for o-xylene oxidation was morphology sensitive^23^. Over the CeO_2_ nanocubes enclosed by (100) planes, water vapor enhanced o-xylene oxidation. As for CeO_2_ nanorods and nanoparticles with predominant exposure of (111) and (100) planes, however, their activities were decreased by water vapor addition. This result possibly indicates that the catalytic activity of (111) planes would be decreased by water vapor addition. With this in mind, opposite changes in o-xylene conversion should be obtained for the samples calcined at 550 °C and 450 °C, if the appearance of new facets in the former one really contributed to the catalytic performance. As shown in Fig. 3, the presence of water vapor always enhanced the catalytic activity of CeO_2_ samples, regardless of the calcination temperature. As for the CeO_2_ calcined at 450 °C and 550 °C, indeed, water vapor addition resulted in increases of 13% and 6% in o-xylene conversion, respectively. These slightly different levels of increase indicate that the contribution of new facets appearing in the sample calcined at 550 °C to o-xylene oxidation was not decisive. It should be noted that observation of the signals assignable to Ce^3+^, O_2_^−^, and Ce^3+^-O^−^Ce^4+^-type defect sites in CeO_2_ is closely related to the introduction and removal of water vapor, which in turn indicates that oxygen vacancies play a key role in the catalytic performance.

Considering a reversible effect of water vapor on the oxygen vacancies, a possible mechanism of oxygen vacancy formation during the calcination process was also proposed. Electron spin resonance measurement revealed that water vapor sharply decreased the intensity of signals relating to oxygen vacancies, while removal of water by heating the sample at the low temperature of 150 °C resulted in the defect sites appearing again. Such reversible effect of water vapor on the oxygen vacancies may provide an important clue to understanding how the calcination process changes the properties of oxygen vacancies on CeO_2_ nanocubes. To test this hypothesis, TG and DSC analyses were performed on an uncalcined sample (Figure S6). This result clearly showed that water desorption and condensation of hydroxyl groups occurred^61^, leading to the formation of oxygen vacancies on the CeO_2_. In agreement with the result of Natile and co-workers, a continuous weight loss due to water desorption and condensation of hydroxyl groups was observed at temperatures below 700 °C, indicating that the higher the calcination temperature, the higher the concentration of oxygen vacancies.

Driven by a gain in energy during agglomeration of single oxygen vacancies or oxygen vacancy clusters with small size, previous studies revealed that oxygen vacancies on the ceria surface are prone to form clusters^42–44^. Using high-resolution scanning tunneling microscopy (STM), Esch *et al*. confirmed that the direct diffusion of oxygen vacancies (i.e. hopping of lattice oxygen) on the CeO_2_ surface requires temperatures higher than 400 °C. With this in mind, we can deduce that the direct diffusion of oxygen vacancies can hardly occur if the samples are calcined at the temperature of 350 °C, thus giving a low possibility for gathering single oxygen vacancies together into a cluster. As a result, the oxygen vacancies on this sample are mainly present as single oxygen vacancies. Calcination the samples at 450, 550, and 650 °C triggered the diffusion of oxygen vacancies, thus oxygen vacancies clusters were predominant on these three samples. Such diffusion possibly became more pronounced with increasing temperature, particularly during calcination at higher temperatures such as 650 °C, leading to oxygen vacancy clusters with bigger size (Table 3). As a result, it is reasonable that the CeO_2_ nanocubes calcined at different temperatures exhibit oxygen vacancies with different sizes and size distributions.

In summary, CeO_2_ nanocubes calcined at 550 °C exhibited the highest activity for catalytic oxidation of o-xylene among the CeO_2_ samples calcined at 350, 450, 550, and 650 °C. The reason was that CeO_2_ samples calcined at 550 °C had oxygen vacancy clusters with suitable size and distribution, which are essential for catalytic reaction via simultaneous adsorption and activation of O_2_ and o-xylene molecules. Water vapor enhanced the complete oxidation of o-xylene through the formation of ·OH radicals, which were helpful for increasing the formation of the intermediate species benzoate, and then promoted the further oxidation of benzoate species into CO_2_, thus increasing the o-xylene conversion and CO_2_ yield.

## Methods

### Catalyst preparation

CeO_2_ nanocubes were synthesized by a hydrothermal method as described in our previous studies^23,62^. Briefly, Ce(NO_3_)_3_·6H_2_O was dissolved in deionized water, and then a suitable amount of 1 mol/L NaOH solution was added dropwise into the above solution. After 20 min of stirring, the mixture was then hydrothermally treated at 100 °C for 12 h. The fresh white precipitates was separated by centrifugation, and thoroughly washed with deionized water. The solid obtained was dried at 60 °C in air for 24 h and calcined at 350, 450, 550 and 650 °C for 4 h in air, respectively.

### Catalytic activity test and kinetic measurements

The catalytic activity was evaluated in a fixed-bed quartz reactor with 100 mg of CeO_2_ nanocubes (40–60 mesh) by passing a reaction gas of 500 ppm o-xylene and 20 vol% O_2_ in N_2_ at a rate of 100 mL/min. The o-xylene gas was supplied by bubbling liquid o-xylene with N_2_, the concentration of which was controlled by the flow rate of N_2_. Analysis of the concentrations of reactants and the products was carried out on-line using a GC-MS (Agilent 6890-5973N) with a HP-5MS capillary column and another GC (GC112A, Shangfen, China). The conversion of o-xylene (*X*_*o*-xylene_, %) and CO_2_ yield (%) were calculated as the following:

4 $${{\rm{X}}}_{o \mbox{-} xylene}=({{\rm{C}}}_{o \mbox{-} \mathrm{xylene}(\mathrm{in})}-{{\rm{C}}}_{o \mbox{-} \mathrm{xylene}(\mathrm{out})})/{{\rm{C}}}_{o \mbox{-} \mathrm{xylene}(\mathrm{in})}\times 100 \% $$

5 $${{\rm{CO}}}_{2}{\rm{yield}}( \% )=[{{\rm{CO}}}_{2}]/{{\rm{8C}}}_{o \mbox{-} xylene(in)}\times 100 \% $$

where C_o-xylene (in)_ (ppm) and C_o-xylene (out)_ (ppm) are the concentrations of o-xylene in inlet and outlet gas streams, respectively. [CO_2_] is the concentration of CO_2_ (ppm).

Kinetic measurements for o-xylene oxidation (reaction rates and orders) were performed at 230 °C by using 100 mg catalyst (40–60 mesh). To realize a differential-reactor assumption, o-xylene conversion was kept below 15% by changing the gas hourly space velocity in the range of 2.3 × 10^5^–1.3 × 10^6^ mL·h^−1^·g^−1^.

### Catalyst characterization

The BET surface areas of CeO_2_ materials were analyzed by N_2_ adsorption at 77 K on a Quantasorb-18 automatic system. XRD patterns were measured on a PANalytical X′Pert PRO X-ray diffractometer (Japan) with a CuKα radiation. TEM observations were carried out on a Hitachi H-7500. The HR-TEM images and SAED patterns were obtained on JEOL JEM 2011 TEM. Raman spectra were measured on a UV Resonance Raman Spectrometer (UVR DLPC-DL-03) equipped with a CCD detector. XPS spectra were measured with a scanning X-ray microprobe (PHI Quantera, ULVAC–PHI, Inc) using Al Kα radiation. Electron Spin Resonance (ESR) was recorded using a Bruker A300-10/12 ESR spectrometer at room temperature. In order to determine the formation of ∙OH radicals in the presence of water vapor, the sample for ESR measurement was also prepared by adding the CeO_2_ to 5,5-dimethyl-1-pirroline-N-oxide (DMPO) solution to form an aqueous dispersion to test for the presence of DMPO-∙OH. PAS measurements were carried out with a magnetically guided variable-energy (0–20 KeV) positron beam and the corresponding results were analyzed with the POSITRONFIT-88 program.

### *In situ* diffuse reflectance infrared Fourier transform spectroscopy (*In situ* DRIFTS)

*In situ* DRIFTS experiments were carried out on a Nicolet Nexus 670 FTIR equipped with a mercury cadmium telluride (MCT) detector, which was cooled by liquid nitrogen. All spectra were measured with a resolution of 4 cm^−1^ and with an accumulation of 100 scans.

## Electronic supplementary material

Supplementary Information
