# Supplementary material for: Oxygen vacancy clusters essential for the catalytic activity of CeO2 nanocubes for o-xylene oxidation
Source: Sci Rep. 2017 Oct 9;7:12845. doi: 10.1038/s41598-017-13178-6 (PMC5634409; doi:10.1038/s41598-017-13178-6)
Supplement: Supplementary file 1 — Supplementary Information [file 41598_2017_13178_MOESM1_ESM.doc]

Electronic Supplementary Information for:

Oxygen vacancy clusters essential for the catalytic activity of CeO_2_ nanocubes for o-xylene oxidation

Lian Wang^1^, Yunbo Yu^1,2^ , Hong He^1,2^, Yan Zhang^1^, Xiubo Qin^3^ & Baoyi Wang^3^

^1^State key Joint Laboratory of Environmental Simulation and Pollution Control, Research Center for Eco-Environmental Sciences, Chinese Academy of Sciences, Beijing 100085, China

^2^Center for Excellence in Regional Atmospheric Environment, Institute of Urban Environment, Chinese Academy of Sciences, Xiamen 361021, China

^3^Key Laboratory of Nuclear Analysis Techniques, Institute of High Energy Physics, Chinese Academy of Sciences, Beijing 100049, China

Correspondence and requests for materials should be addressed to Y.Y. and H.H. (email: ybyu@rcees.ac.cn; honghe@rcees.ac.cn)

**List of contents**

**Figure S1**. XRD patterns of the CeO_2_ nanocubes calcined at different temperatures.

**Figure S2**. Raman shift of the CeO_2_ nanocubes calcined at different temperatures.

**Figure S3**. HRTEM images of CeO_2_ nanocubes calcined at different temperatures.

**Figure S4.** Ce3d XPS spectra of CeO_2_ nanocubes calcined at different temperatures (a); the possible individual contributions (XPS spectra of CeO_2_ calcined at 550 ^o^C) (b) (Peaks labeled by subscripts ν and u are contributed to 3d5/2 and 3d3/2 states, respectively); O1s XPS spectra of CeO_2_ nanocubes calcined at different temperatures (c).

**Figure S5.** The lifetime spectra of PAS of CeO_2_ nanocubes calcined at different temperatures.

**Figure S6.** Thermo-gravimetric **(**TG) spectra of CeO_2_ nanocubes calcined with a heating rate of 10 ^o^C/min.

**Table S1.** XPS binding energies of individual peaks of the Ce 3d spectra for CeO_2_ nanocubes calcined at different temperatures.

**Table S2.** Assignment of the *in situ* DRIFTS bands.

**
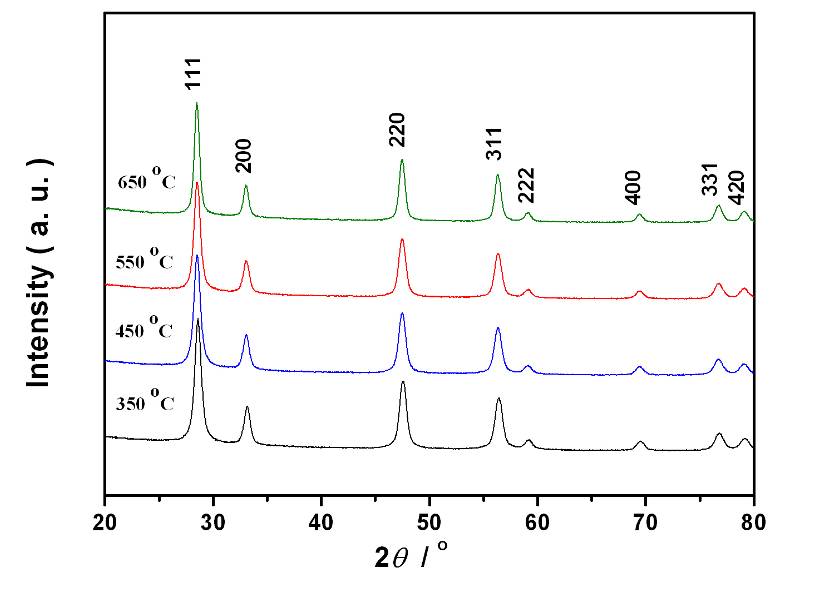
**

**Figure S1**. XRD patterns of the CeO_2_ nanocubes calcined at different temperatures.


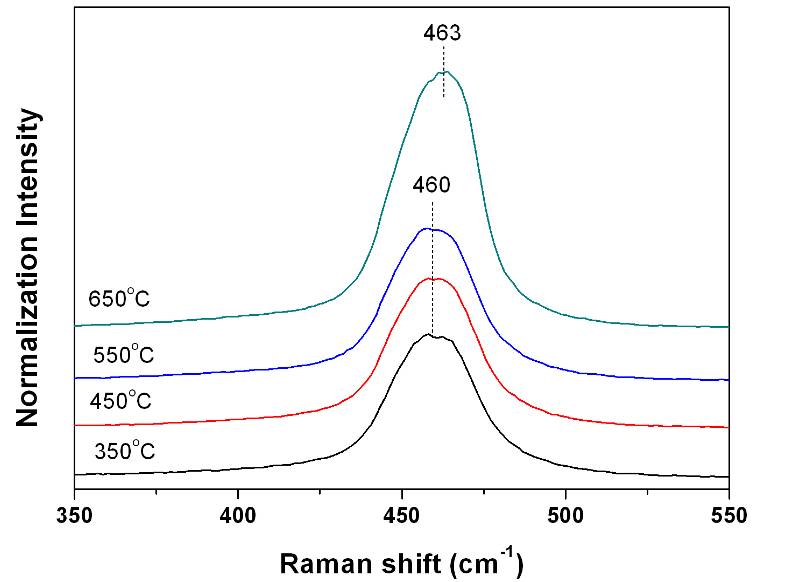


**Figure S2**. Raman shift of the CeO_2_ nanocubes calcined at different temperatures.

Raman spectra of CeO_2_ nanocubes calcined at different temperatures are shown in Figure S2. A strong peak at 460 cm^-1^ corresponding to the triply degenerate F_2g_ mode of fluorite CeO_2_ (the only one allowed in first order) was observed for the samples calcined within the temperature range of 350-550 ^o^C, indicating that these samples exhibited the same particle size.^1^ However, the sample calcined at 650 ^o^C exhibited the F_2g_ mode feature at 463 cm^-1^, confirming an increase of the particle size of CeO_2_.


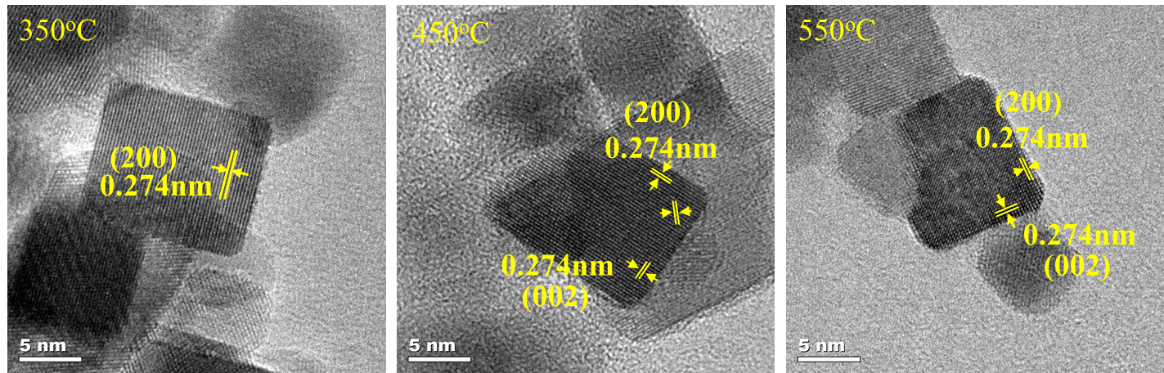


**Figure S3**. HRTEM images of CeO_2_ nanocubes calcined at different temperatures.

Figure S3 shows crystal lattice images of some typical CeO_2_ nanocubes calcined at temperatures of 350-550 ^o^C. The HRTEM image combined with FFT analysis (Figure 1f) displays the clear (200) and (220) lattice fringes with the interplanar spaces of 0.274 and 0.189 nm, respectively, generally implying that the CeO_2_ nanocubes are enclosed by (100) planes.^2^


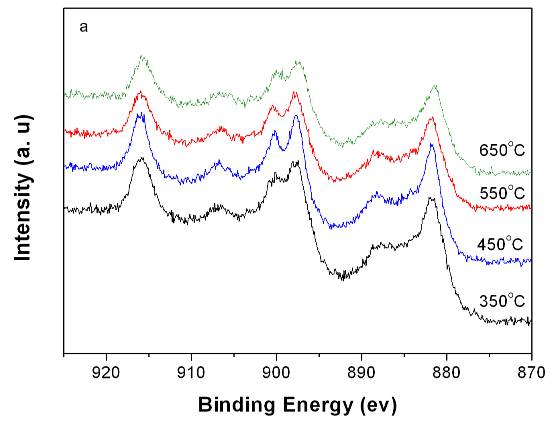

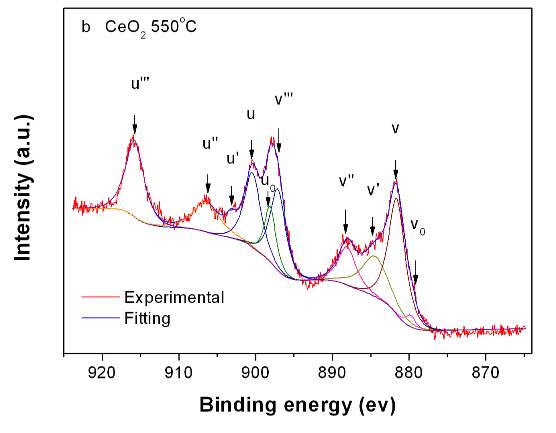


**Figure S4.** Ce3d XPS spectra of CeO_2_ nanocubes calcined at different temperatures (a); the possible individual contributions (XPS spectra of CeO_2_ calcined at 550 ^o^C) (b) (Peaks labeled by subscripts ν and u are contributed to 3d5/2 and 3d3/2 states, respectively); O1s XPS spectra of CeO_2_ nanocubes calcined at different temperatures (c).

Figure S4a shows Ce3d XPS spectra of CeO_2_ nanocubes calcined at different temperatures, together with the corresponding peak fitting results. The ν_0_, ν’, u_0_, and u’ peaks are corresponding to Ce^3+^; while ν, ν’’, ν’’’, u, u’’, u’’’ are contributed to Ce^4+^.^3,4^ The peak positions for all the samples calcined at different temperatures are listed in Table S1.

**Table S1.** XPS binding energies of individual peaks of the Ce 3d spectra for CeO_2_ nanocubes calcined at different temperatures.

| Calcination Temperature (^o^C) | Ce^4+^ | | | | | | Ce^3+^ | | | |
| --- | --- | --- | --- | --- | --- | --- | --- | --- | --- | --- |
|  | ν | ν” | ν"’ | u | u” | u”’ | ν_0_ | ν' | u_0_ | u' |
| 350 | 881.6 | 887.8 | 897.4 | 900.3 | 906.6 | 915.7 | 879.7 | 884.6 | 898.3 | 902.9 |
| 450 | 881.6 | 887.8 | 897.3 | 900.3 | 906.6 | 915.9 | 879.5 | 884.2 | 898.2 | 903.0 |
| 550 | 881.6 | 888.1 | 897.1 | 900.4 | 906.4 | 915.7 | 879.8 | 884.4 | 898.1 | 903.2 |
| 650 | 881.6 | 888.0 | 897.1 | 900.2 | 906.4 | 915.5 | 879.8 | 884.5 | 898.2 | 903.2 |

The Ce^3+^ concentration in CeO_2_ nanocubes calcined at different temperatures was calculated by analysis of the integrated peak area, with the equation shown as following:

$$[Ce^{3+}]=\frac{A_{\upsilon0}+A_{\upsilon'}+A_{u0}+A_{u'}}{A_{\upsilon0}+A_{\upsilon'}+A_{u0}+A_{u'}+A_{\upsilon}+A_{\upsilon''}+A_{\upsilon'''}+A_{u}+A_{u''}+A_{u'''}}$$

where A_i_ is the integrated area of peak “i”.

**Figure S5.** The lifetime spectra of CeO_2_ nanocubes calcined at different temperatures.

**Table S2.** Assignment of the *in situ* DRIFTS bands.

| Wavenumber (cm^-1^) ^in this study^ | Assignment | Characteristic of | Wavenumber (cm^-1^)^5^ | Wavenumber (cm^-1^)^6^ |
| --- | --- | --- | --- | --- |
| 3067, 3031 | phenylic C-H vibration | aromatic ring | 3075, 3033 | 3073, 3034 |
| 2948 | C-H symmetric vibration | aromatic ring | 2932 | 2934 |
| 2878 | C-H antisymmetric vibration | aromatic ring | 2877 | 2883 |
| 1604 | skeletal C-C vibration | aromatic ring | 1596 | 1607-1601 |
| 1546 | C-O antisymmetric vibration | benzoate | 1540 | 1566-1554 |
| 1468 | skeletal C-C vibration | aromatic ring | 1501 | 1497 |
| 1396 | C-O symmetric vibration | benzoate | 1425-1412 | 1415 |

**Figure S6** Thermo-gravimetric **(**TG) spectra of CeO_2_ nanocubes calcined with a heating rate of 10 ^o^C/min.

**References**

1. Spanier, J. E., Robinson, R. D., Zhang, F., Chan, S.-W. & Herman, I. P. Size-dependent properties of CeO_2-y_ nanoparticles as studied by Raman scattering. *Phys. Rev. B* **64**, 245407 (2001).
2. Mai, H. X. *et al*. Shape-selective synthesis and oxygen storage behavior of ceria nanopolyhedra, nanorods, and nanocubes. *J. Phys. Chem. B* **109***,* 24380–24385 (2005).
3. Xu, J. *et al*. Size dependent oxygen buffering capacity of ceria nanocrystals. *Chem. Commun.* **46**, 1887–1889 (2010)
4. Liu, X. W., Zhou, K. B., Wang, L., Wang, B. Y. & Li, Y. D. Oxygen vacancy clusters promoting reducibility and activity of ceria nanorods. *J. Am. Chem. Soc.* **131**, 3140–3141 (2009).
5. Besselmann, S., Löffler, E. & Muhler, M. On the role of monomeric vanadyl species in toluene adsorption and oxidation on V_2_O_5_/TiO_2_ catalysts: a Raman and in situ study. *J. Mole. Catal. A: Chem.* **162**, 401–411 (2000).
6. Zhao, S., Li, K. Z., Jiang, S. & Li, J. H. Pd–Co based spinel oxides derived from Pd nanoparticles immobilized on layered double hydroxides for toluene combustion. *Appl. Catal. B* **181**, 236–248 (2016).
